# Therapeutic Intervention of Neuroinflammatory Alzheimer Disease Model by Inhibition of Classical Complement Pathway with the Use of Anti-C1r Loaded Exosomes

**DOI:** 10.21203/rs.3.rs-3399248/v1

**Published:** 2023-10-18

**Authors:** Terjahna Richards, Jeanette C. Perron, Ketan Patel, John Wurpel, Sandra E. Reznik, Francis Schanne

**Affiliations:** St. John’s University; St. John’s University; St. John’s University; St. John’s University; St. John’s University; St. John’s University

**Keywords:** Complement inhibitors, exosome, neuroinflammation

## Abstract

Alzheimer’s disease (AD) is a complex neurodegenerative disease associated with memory decline, cognitive impairment, amyloid plaque formation and tau tangles. Neuroinflammation has been shown to be a precursor to apparent amyloid plaque accumulation and subsequent synaptic loss and cognitive decline. In this study, the ability of a novel, small molecule, T-ALZ01, to inhibit neuroinflammatory processes was analyzed. T-ALZ01, an inhibitor of complement component C1r, demonstrated a significant reduction in the levels of the inflammatory cytokines, IL-6 and TNF-α *in vitro*. An LPS-induced animal model, whereby animals were injected intraperitoneally with 0.5 mg/kg LPS, was used to analyze the effect of T-ALZ01 on neuroinflammation *in vivo*. Moreover, exosomes (nanosized, endogenous extracellular vehicles) were used as drug delivery vehicles to facilitate intranasal administration of T-ALZ01 across the blood-brain barrier. T-ALZ01 demonstrated significant reduction in degenerating neurons and the activation of resident microglia and astrocytes, as well as inflammatory markers *in vivo*. This study demonstrates a significant use of small molecule complement inhibitors via exosome drug delivery as a possible therapeutic in disorders characterized by neuroinflammation, such AD.

## Introduction

Alzheimer’s disease (AD) is a global health and socioeconomic concern, contributing to one of the most common causes of dementia [1], [2]. Currently, over 50 million people are globally affected with AD, and this is expected to rise to 152 million people by 2050 [1], [2]. AD is a multifactorial and polygenic neurodegenerative disease that begins in the hippocampus and cerebral cortex and is characterized by cognitive decline and memory loss. Major hallmarks of AD include β-amyloid (Aβ) plaque formation, neurofibrillary tangles (NFTs) and neuroinflammation [1], [2]. Over the last decade, few FDA-approved therapeutics have been established for the treatment of AD including acetylcholinesterase inhibitors (donepezil, rivastigmine) and non-competitive N-methyl-D-aspartate antagonists (memantine). Most recently, monoclonal anti-amyloid therapeutics – aducanumab and lecanemab – have been developed for the treatment of this neurodegenerative disease [3], [4]. However, the associated possibility of brain swelling increases health and financial burden on AD patients [3], [5], [6]. The success rate of AD drugs is low, stemming from the complex pathophysiology and a poor understanding of the disease progression.

The role of neuroinflammation in various neurodegenerative diseases, including AD, has been alternatively considered. Epidemiological evidence of neuroinflammation is demonstrated in the protective use of non-steroidal anti-inflammatory drugs (NSAIDs) and moderate reduction in the risk of AD [7]–[9]. Furthermore, genetic and biofluid markers indicative of neuroinflammation were shown to increase in AD [10], [11]. Neuroinflammation is also present in AD patients regardless of Aβ load, indicating a possible initiator role for neuroinflammation in the development of AD [12]–[14].

Neuroinflammation occurs in the central nervous system (CNS) in response to several factors such as CNS injury, infections, toxins, or autoimmunity [13], [14]. The duration, type and actions of inflammatory factors can further determine the severity of the neuroinflammatory response. Neuroinflammation plays both a protective and pathological role in the CNS. It is important for brain development, tissue repair after injury, brain plasticity and restoration. However, neuroinflammation can lead to cognitive impairment, neuronal damage, reduced plasticity, anxiety, and depression. The inflammatory response is mediated by the cells of the CNS through the production of cytokines, chemokines, reactive oxygen species and other messengers that contribute to several pathways [15], [16]. However, targeting these inflammatory pathways have not yielded effective AD therapeutics and thus, investigators have begun to consider the role of the inflammatory Pathways of Complement Fixation in the development of AD [17]–[21].

The complement system is comprised of over 40 proteins that play a dominant role in initial activation of an inflammatory response and, antibody-mediated pathogen opsonization and clearance [18], [19]. The complement system is activated by three different recognition pathways (classical, alternative and lectin) which lead to sequential enzyme activation, protein cleavage and induced function-enabling protein conformational changes. Regardless of the activation mechanism, the three major pathways result in: (1) opsonization, via the deposition of the activation dependent cleavage fragments, which tag pathogens for more efficient phagocytosis, (2) leukocyte/microglia recruitment to the site of injury via production of chemotactic peptides and (3) targeted death of pathogens due to the creation of a membranolytic pore in pathogen cell membranes [17], [22], [23]. The generation of the diffusible chemotactic peptides can also lead to the alteration of immune (and other) cellular activities such as production of reactive oxidation species and secretion of pro-inflammatory cytokines that have evolved to aid in the efficient clearance of pathogens. The complement system is normally controlled by several circulating (C1 inhibitor, factor H, C4b-binding protein) and membrane-associated (CD46, CD55, CR1, CD59) complement regulators [17], [19], [23]. However, in AD, the excessive activation and poor regulation of the complement system can lead to neurodegeneration, cognitive decline, and memory loss [17], [19].

Numerous small-molecule anti-complement drugs are in development or currently in use for several neurodegenerative and inflammatory diseases such as AD [17]–[19], [22], [23]. Of note, three drugs are currently licensed for use in inflammatory diseases – C1INH (Cinryze, Berinert), C5 monoclonal antibody (Eculizumab) and FUT-175. C1INH, similar to the intrinsic C1 inhibitor, reduces duration and severity of acute immune attacks and Eculizumab prevents MAC formation [18], [19]. The small molecule FUT-175 is a potent serine protease inhibitor that affects enzymatic activities of C1r, C1s, factor D and C3/C5 [18]. However, the off-target effects of these molecules result in toxicity, making them poor candidates for clinical use as a therapeutic in AD.

Analysis of the complement pathway has regarded the initiating complex of the classical complement pathway as a possible therapeutic target [24], [25]. Research by Hong *et al*. analyzed the efficacy of an anti-C1q inhibitor for the treatment of neuroinflammation [25]. Results demonstrated a reduction in neuroinflammation but the inhibitor of C1q also reduced opsonization and immune clearance [25]. In 2020, Rushing *et al*. discovered two small molecules (CMP-1611 and CMP-1696) that selectively inhibit C1r through fragment-based discovery analysis [26]. C1r consists of two N-terminal CUB domains, an epidermal growth factor (EGF)-like domain, two complement control modules (CCP) and a C-terminal serine protease (SP) domain. Though the mechanism of action of CMP-1611 is unknown, CMP-1696, referred to as T-ALZ01, was found to competitively inhibit C1r through binding of the CCP2-SP region, a functional region conserved in different species such as humans, mice, and rats [26]. Though T-ALZ01 is a possible therapeutic, its inability to cross the blood-brain barrier warranted the use of an alternative method of drug delivery. Exosomes are nanosized, extracellular vesicles characterized by a lipid bilayer encompassing biological components [27]–[29]. Exosomes demonstrate specific targeting capabilities to the cell type from which they were derived, thus, microglia derived exosomes were utilized to ensure targeted delivery of T-ALZ01 to the microglia [27]–[29]. Exosomes as a drug delivery tool would prove beneficial in reducing economic and social burden, making the administration of therapeutic easier on patient and doctor.

The primary objective of this study focuses on the hypothesis that T-ALZ01, loaded into microglia-derived exosomes, would inhibit the inflammatory processes that cause neurodegeneration and interrupt cellular communication in AD. Given the mechanism of action and specificity of T-ALZ01, several inflammatory markers were assessed in an *in vitro* and *in vivo* neuroinflammatory model of AD where the candidate drug was delivered by microglia-derived exosomes. T-ALZ01 demonstrated promising results as a potential AD therapeutic through the reduction of inflammatory markers.

## Results

### T-ALZ01 demonstrates low IC_50_ in SD cells exposed to LPS

To first determine how T-ALZ01 affects primary Sprague Dawley (SD) microglia cells and to establish the range of subtoxic concentrations for future studies, the MTT cell viability assay was carried out. Primary SD microglia cells were exposed to increasing concentrations (5–500 μM) of T-ALZ01 for 72 hours. Donepezil (DPZ), an FDA approved therapeutic in neuroinflammatory conditions, was used to compare the efficacy of T-ALZ01. Following the 72-hour exposure period, the MTT cell viability substrate was added to the cultures. Only metabolically active cells can reduce the substrate and, thereby, generate a product that allows for the measurement of absorbance. Any decrease in absorbance was considered an indication of cell death or toxicity. In contrast, increases in absorbance readings are typically due to proliferation of cells in the treated cultures.

**Figure 1:**
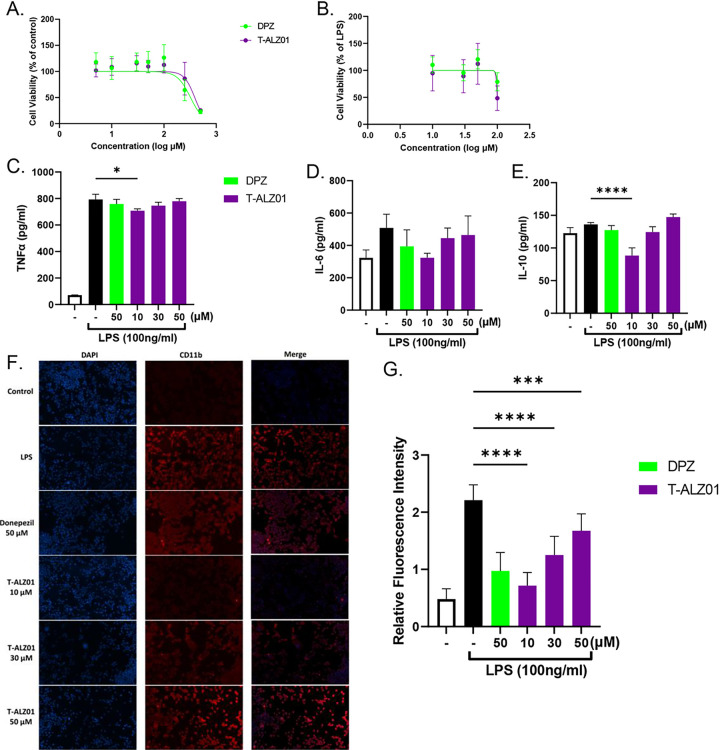
T-ALZ01 significantly reduced neuroinflammation in SD microglia cells. Primary SD microglia cells were exposed to LPS (100 ng/mL) followed by treatment with T-ALZ01 (10, 30 and 50 μM) for 72 hours. DPZ (50 μM) was used to compare efficacy of T-ALZ01. Cell viability is presented as a percentage of absorbance detected in control, untreated cells **(A)** or in LPS-treated cells **(B)**. Non-linear regression analysis revealed an IC_50_ value of 388.8 μM in control untreated cells **(A)** and an IC_50_ value of 99.8 μM in LPS-treated cells **(B)**. **(C-E)**. Analysis of inflammatory cytokine levels showed a decrease in IL-6 but significant decline in TNF-α and IL-10 following treatment with 10 μM T-ALZ01 compared with LPS-only group (*p < 0.05; and ****p < 0.001, respectively). **(F-G)**. SD microglia cells grown on PDL-coated glass coverslips were exposed to LPS (100 ng/mL) followed by treatment with DPZ (50 μM) or T-ALZ01 (10, 30 and 50 μM) for 72 hours. Untreated cells served as the negative control. The cultures were fixed and labelled with anti-CD11b antibodies and Cy3-conjugated secondary antibodies (red). The coverslips were mounted in medium containing DAPI to label nuclei (blue). Control cells have low levels of CD11b compared to the LPS-only group (****p < 0.001). **(G)** Treatment with T-ALZ01 (10 μM) resulted in a drastic decrease in CD11b expression compared to the LPS-only group (****p < 0.001). The merged panels **(F)** illustrate the complete overlap of CD11b labelling and the DAPI stain (n=3; scale =200 μm).

A non-linear curve analysis was carried out to calculate the IC_50_ value allowing determination of the concentration of T-ALZ01 at which the cell viability is reduced by 50%. The IC_50_ value for T-ALZ01 was 388.8 μM (Fig. 1A). These results indicate that concentrations of T-ALZ01 from 5 μM to 100 μM are non-toxic under healthy conditions. To further investigate the effect of T-ALZ01 in an LPS-induced neuroinflammatory environment, the SD microglial cells were exposed to 100 ng/mL LPS. The LPS was added 30 minutes prior to the addition of T-ALZ01. In a neuroinflammatory environment, T-ALZ01 demonstrated a IC_50_ value of 99.83 μM (Fig. 1B). The lower IC_50_ observed after exposure to LPS indicates a more sensitive response to T-ALZ01. Taken together, these data demonstrate that T-ALZ01 is safe to use at concentrations at 10–50 μM. These experiments were also conducted in an immortalized mouse cell line (SIM-A9) which yielded lowered IC_50_ after LPS exposure (IC_50_ = 326.7, healthy; IC_50_ = 33.26, LPS) (Supplementary Figure S1A-B).

### Low concentration of T-ALZ01 reduces neuroinflammation in SD microglia cells

To ascertain whether this novel therapeutic can alleviate neuroinflammation in primary SD microglia cells, the levels of pro-inflammatory (TNF-α and IL-6) and anti-inflammatory (IL-10) cytokines were assessed in response to T-ALZ01. The SD microglia cells were exposed to 100 ng/mL LPS followed by T-ALZ01 (10, 30 and 50 μM) or DPZ (50 μM) for 72 hours. Enzyme-linked immunosorbent assay (ELISA) of the supernatant of SD microglia cells showed that T-ALZ01 was most effective at reducing neuroinflammatory cytokines at low concentrations. T-ALZ01 (10 μM) decreased the levels of pro-inflammatory TNF-α (*p < 0.05) and IL-6 compared to the LPS-only group (Fig. 1C–D, respectively). Anti-inflammatory IL-10 was significantly decreased compared to the LPS-only group (****p < 0.001) and compared to the untreated cells (**p < 0.01) (Fig. 1E). Similar tests conducted in SIM-A9 microglia cells demonstrated greater efficacy of DPZ, compared to T-ALZ01, in the reduction of neuroinflammatory cytokines (Supplementary Fig. 1C-E). T-ALZ01 (10, 30 and 50 μM) demonstrated no statistically significant differences compared to the LPS-only group (Supplementary Figure S1C-E).

To establish that T-ALZ01 also decreases microglia activation, indicated by CD11b expression levels, in an LPS-induced neuroinflammatory environment, immunofluorescent labeling was performed. Primary SD microglia cells grown on poly-D-lysine (PDL)-coated coverslips were exposed to LPS (100 ng/mL) for 30 minutes followed by treatment with DPZ (50 μM) or T-ALZ01 (10, 30 and 50 μM) for 72 hours. The cultures were fixed and labelled with anti-CD11b antibodies (red) and the nuclei stain, DAPI (blue) (Fig. 1F). In control, unstimulated SD microglia cells, low expression levels of CD11b were detected (Fig. 1F–G). The LPS-only group induced significantly greater CD11b expression compared to the control, untreated cells (****p < 0.001) (Fig. 1F–G). T-ALZ01 (10 μM) demonstrated the most significant decrease of CD11b expression compared to the LPS-only group (**** p < 0.001) (Fig. 1F–G). Thus, T-ALZ01 (10 μM) not only strongly reduces inflammatory cytokine levels but also decreases CD11b expression in SD microglia cells. In contrast, SIM-A9 microglia cells demonstrated strong CD11b expression despite significant decrease in expression levels compared to LPS-only group (****p < 0.001) (Supplementary Figure S1F-G).

### T-ALZ01 affects the complement pathway in a dose-dependent manner in SD microglia cells

**Figure 2:**
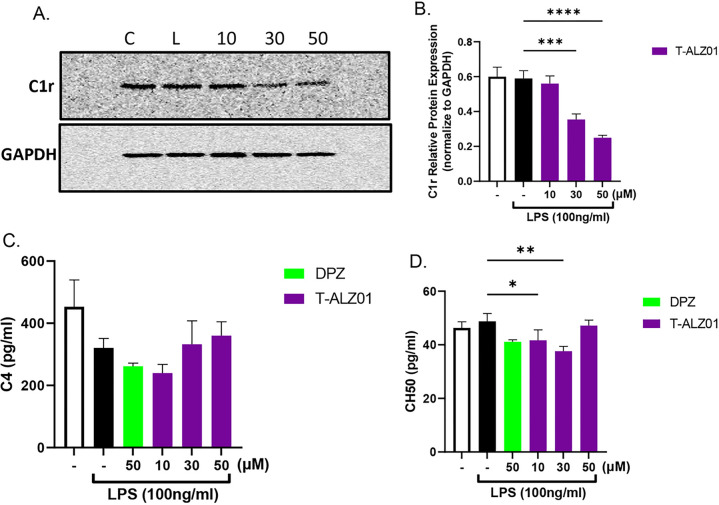
T-ALZ01 affects the complement pathway in a dose-dependent manner in SD microglia cells. Primary SD microglia cells were exposed to LPS (100 ng/mL) followed by treatment with T-ALZ01 at 10, 30 and 50 μM. Western blot analysis using anti-C1r antibodies showed that the concentration of T-ALZ01 decreased the expression of C1r in a dose-dependent manner **(A)**. **(B)** Quantification of C1r levels compared to the LPS-only group confirms significant downregulation of C1r following exposure to increasing concentrations of T-ALZ01 (10 μM: no significance; 30 μM: ***p < 0.005; 50 μM: ****p < 0.001). Levels of C1r were normalized to GAPDH and are expressed as a percent of control (mean ± SD; n=3). **(C-D)** ELISA analysis of the expression of complement protein C4 and complement hemolysis 50% (CH50) demonstrated significant reduction in protein expression of C4 (**p < 0.01, compared to control) and CH50 (*p < 0.05, compared to LPS-only) following treatment with 10 μM T-ALZ01. Data are expressed as mean ± SD (n=3) with each experiment performed in triplicate.

To investigate the effect of T-ALZ01 on the expression of C1r in LPS-induced neuroinflammation, the levels of C1r in primary SD microglia cells treated with T-ALZ01 (10, 30 and 50 μM) was assessed by Western blot analysis (Fig. 2A–B). Whole cell lysates of treated SD microglia cells were probed for C1r using an anti-C1r antibody. C1r levels were normalized against GAPDH. The LPS-only microglia cells served as positive control and unstimulated cells helped to establish the baseline levels of C1r. T-ALZ01 exhibited a dose-dependent inhibitory response. The higher the concentration of the drug, the lower the expression of C1r. Though there was no statistical significance between the LPS-only group and 10 μM T-ALZ01, significant reduction of C1r was observed in 30 μM (***p < 0.005) and 50 μM (****p < 0.0001) compared to LPS-only (Fig. 2A–B). Western blot analysis of the effect of T-ALZ01 on the expression of C1r in SIM-A9 microglia cells were also conducted and demonstrated a similar dose-dependent inhibitory effect compared to the LPS-only group (10, 30 and 50μM, ****p < 0.001) (Supplementary Figure S2A-B).

To establish T-ALZ01 effect on the complement pathway, ELISA of complement protein C4 and the complement hemolysis 50% (CH50) in SD microglia cells (Fig. 2C–D). Primary SD microglia cells exposed to 100 ng/mL LPS were treated with T-ALZ01 (10, 30 and 50 μM) for 72 hours. In contrast to the expression of C1r, the 10 μM T-ALZ01 demonstrated a significant decrease of the protein levels of C4 (**p < 0.01) and CH50 (*p < 0.05) compared to the untreated and LPS groups respectively (Fig. 2C–D). T-ALZ01 also demonstrated a significant decline in CH50 protein levels compared to LPS-only group (**p < 0.01) (Fig. 2C–D).

### Microglia-derived exosomes can be utilized as a drug delivery tool

**Figure 3:**
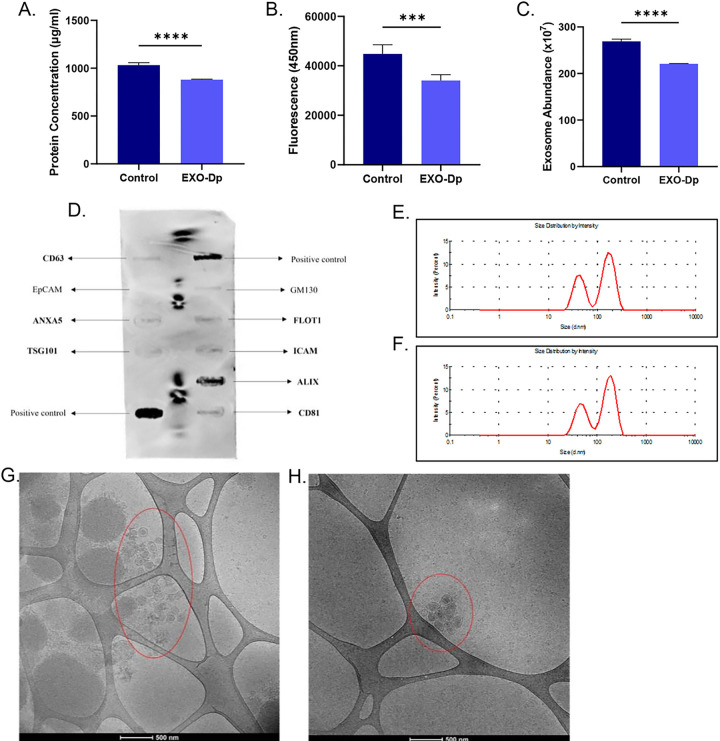
Quantification and characterization of microglia-derived exosomes. SD microglia cells were cultured in normal media (Control) and exosome-depleted FBS-containing media (EXO-Dp) for 5–7 days. The supernatant was collected, and exosomes were isolated using the polyethylene glycol method. **(A-C)** Quantitative analysis of the exosome samples revealed a significant decrease in **(A)** protein concentration (*p < 0.05), **(B)** exosome concentration (***p < 0.005) and **(C)** abundance (****p < 0.001) of the EXO-Dp group compared to the Control group. **(D)** The ExoCheck antibody array confirmed the samples contained exosomes through visualization of bands pre-stained with known exosome markers. **(E-F)** Particle size analysis showed the size of the isolated exosomes in the Control (172.4 nm) and EXO-Dp (182.4 nm) groups. **(G-H)** Cryo-Transmission electron microscopy also demonstrated a larger population of exosomes in the Control group compared to the EXO-Dp group. Data are expressed as mean ± SD (n=3) with each experiment performed in triplicate.

Considering the inability of the novel anti-C1r inhibitor to cross the blood-brain barrier (BBB), microglia-derived exosomes were isolated using the polyethylene-glycol method for use in drug delivery. Exosomes are nanosized, extracellular vesicles that demonstrate a heterogenous population (Supplementary Figure S3). Primary SD microglia cells were cultured in normal media (Control) or exosome-depleted media (EXO-Dp) for 5–7 days and the supernatant was collected for exosome isolation. Quantitative analysis of the exosome samples revealed a significant decrease in protein concentration (*p < 0.05), exosome concentration (***p < 0.005) and abundance (****p < 0.001) of the EXO-Dp group compared to the Control group (Fig. 3A–C, respectively). Characterization of the exosome samples indicated successful isolation of microglia-derived exosomes. The ExoCheck Antibody Array confirmed the sample contained exosomes with a high expression of ALIX, an exosomal marker (Fig. 3D). Particle size analysis showed the size of the isolated exosomes in the Control (172.4 nm) and EXO-Dp (182.4 nm) groups (Fig. 3E–F, respectively). Cryo-Transmission electron microscopy demonstrated a larger population of exosomes in the Control group compared to the EXO-Dp group (Fig. 3G–H, respectively). These results indicate successful isolation of exosomes and removal of the exosomes found intrinsically in FBS. EXO-Dp exosome samples were used in the drug loading process of T-ALZ01. The structure of T-ALZ01 was identified and the success of the drug loading was evaluated using HPLC and LC-MS (Supplementary Figure S4).

### T-ALZ01 reduces neurodegeneration in the hippocampus and cortex of male and female SD rats

**Figure 4:**
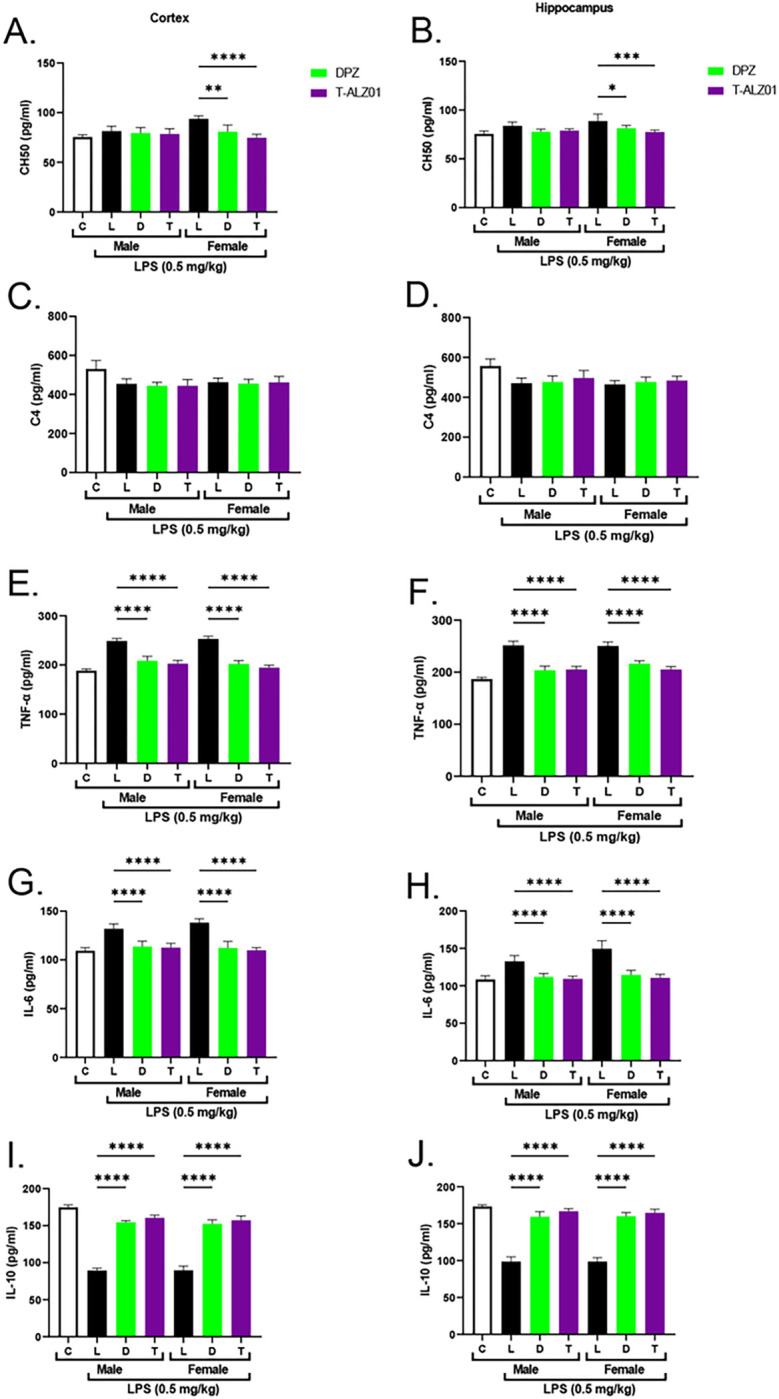
T-ALZ01 reduces neuroinflammation in tissue homogenates of male and female SD rats. One of the mid-sagittal hemisections of the isolated brains from the treated male (n=18) and female (n=18) wild-type SD rats were homogenized and used for the analysis of cytokine protein levels and complement activity. Untreated male SD rats (4-month-old) were used as negative control. **(A-D)** ELISA analysis of complement activity in the tissue homogenates indicated significant reduction in the protein levels of CH50 in the cortex (****p < 0.001) and hippocampus (***p < 0.005) of female SD rats but no statistical significance in the protein levels of C4 for T-ALZ01 or DPZ. No statistical significance observed in the male SD rats. **(E-H)** ELISA analysis of tissue homogenates demonstrated significant decline of the protein levels of TNF-α and IL-6 in the cortex (****p < 0.001) and hippocampus (****p < 0.001) of male SD rats. **(E-H)** Female SD rats revealed a significant decrease in the levels of TNF-α and IL-6 in the cortex (****p < 0.001) and hippocampus (****p < 0.001) following treatment with T-ALZ01 and DPZ. **(I-J)** The levels of the anti-inflammatory IL-10 were significantly increased in male and female in the cortex (****p < 0.001) and hippocampus (****p < 0.001). Data are expressed as mean ± SD (n=6) and each experiment was conducted in duplicate.

To examine the ability of T-ALZ01 to reduce neurodegeneration *in vivo*, an LPS-induced neuroinflammatory model mimicking AD was established in male and female SD rats. The animals were exposed to LPS (1 mg/kg) for 1 hour followed by treatment with DPZ (1 mg/kg) or T-ALZ01 (0.2 mg/kg) every day for 7 days. Phenotypic analysis of the efficacy of T-ALZ01 in reducing neuroinflammation was conducted by counting fecal pellets (Supplementary Figure S5). LPS affects the gut and thus causes frequent bowel movements. The analysis of fecal pellets following treatment can be used to determine the effect of the drug to alleviate LPS-related symptoms. Complement analysis of the CNS tissue homogenates indicated a significant reduction in the protein levels of CH50 was observed in the cortex (****p < 0.001) and hippocampus (***p < 0.005) of female SD rats but no statistical significance in the protein levels of C4 (Fig. 4A–D, respectively). Western blot analysis of the tissue homogenates indicated that the concentration of T-ALZ01 did not significantly reduce the relative protein expression of C1r (Supplementary Figure S6). ELISA analysis of tissue homogenates demonstrated significant decline of the protein levels of TNF-α and IL-6 in the cortex (****p < 0.001) and hippocampus (****p < 0.001) of male SD rats (Fig. 4E–H respectively). Similarly, female SD rats revealed a significant decrease in the levels of TNF-α and IL-6 in the cortex (****p < 0.001) and hippocampus (****p < 0.001) (Fig. 4E–H, respectively). The levels of the anti-inflammatory IL-10 were significantly increased in male and female in the cortex (****p < 0.001) and hippocampus (****p < 0.001) (Fig. 4I–J, respectively).

**Figure 5:**
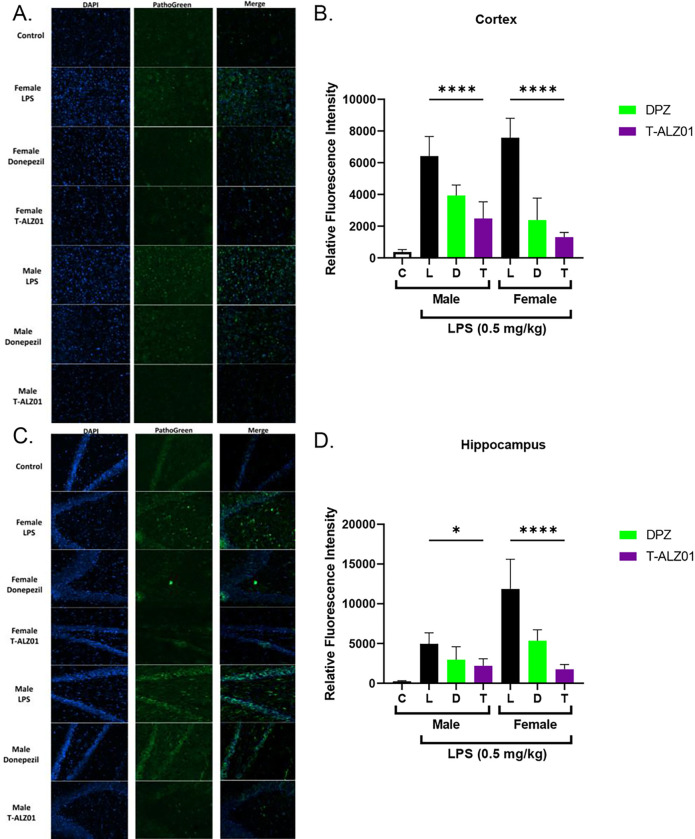
T-ALZ01 reduces neurodegeneration in the cortex and hippocampus of male and female SD rats. One of the mid-sagittal hemisections of the isolated brains from the treated male (n=18) and female (n=18) wild-type SD rats were sectioned (14 μM; n=12–15 slices/group) for analysis of degenerating neurons. Untreated male SD rats (4-month-old) were used as negative control (n=4). The tissue sections were labelled with a histofluorescent anionic dye (green) specific for degenerating neurons and DAPI to label nuclei (blue). **(A)** T-ALZ01 exhibited reduced fluorescence intensity of the anionic dye in male and female SD rats compared to the LPS-only group. **(B)** Quantification of results demonstrated significant decrease of the expression of the anionic dye, compared to the LPS-only group, in the cortex of male (****p < 0.001) and female (****p < 0.001) SD rats. **(C)** T-ALZ01 showed a decrease in the fluorescence intensity of the anionic dye in the hippocampus of male and female SD rats compared to the LPS-only group. **(D)** Quantification of results showed significant reduction of the anionic dye in the hippocampus of male (*p < 0.05) and female (****p < 0.001) SD rats. The merged panels **(A, C)** illustrate the complete overlap of CD11b labelling and the DAPI stain (n=3; scale =200 μm).

**Figure 6:**
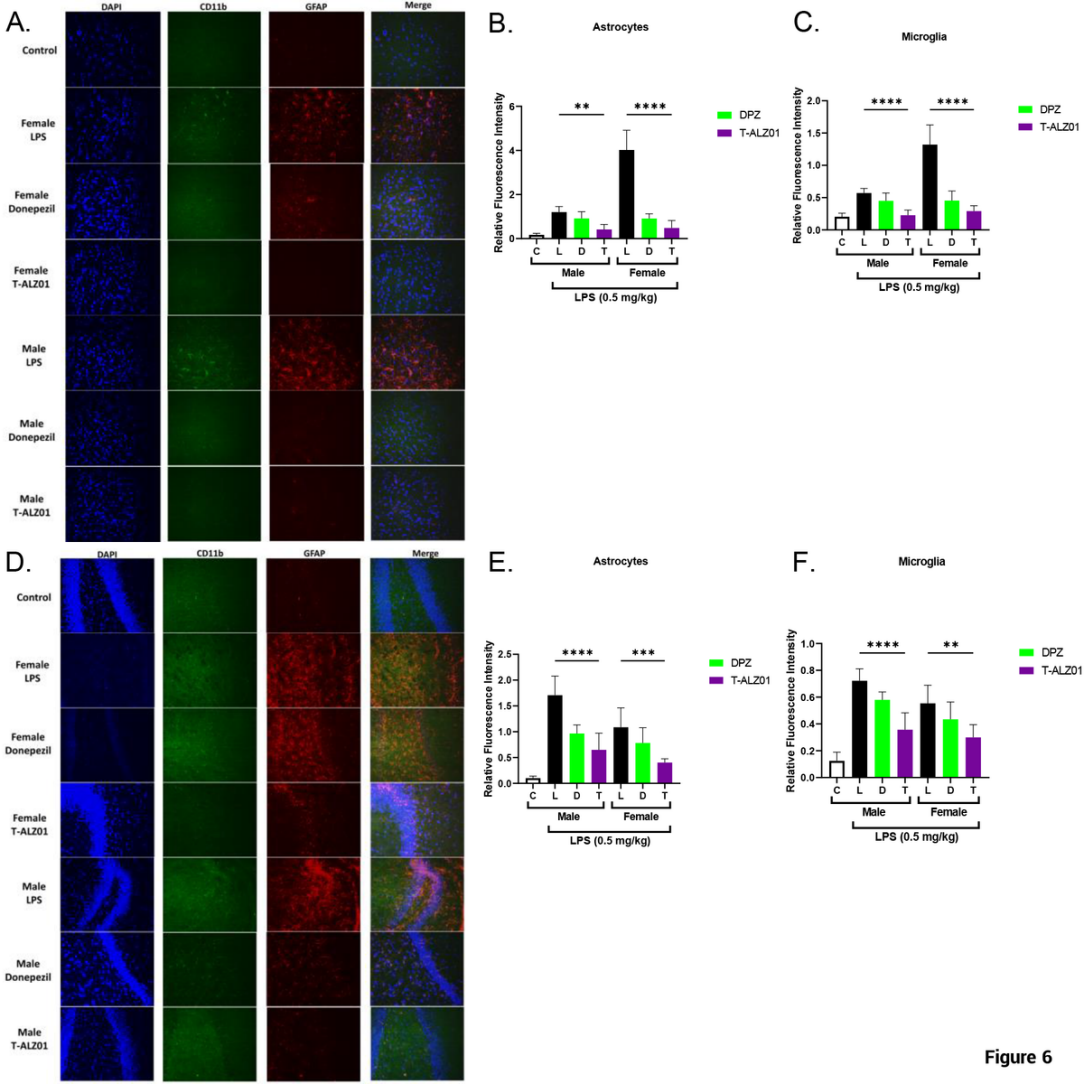
T-ALZ01 decreases the activation of microglia and astrocytes in the cortex and hippocampus of male and female SD rats. One of the mid-sagittal hemisections of the isolated brains from the treated male (n=18) and female (n=18) wild-type SD rats were sectioned (14 μM; n=12–15 slices/group) for analysis of microglia and astrocytes. Untreated male SD rats (4-month-old) were used as negative control (n=4). The tissue sections were labelled with anti-CD11b (green; microglia) and anti-GFAP (red; astrocytes) antibodies as well as the nuclei stain, DAPI (blue). **(A)** T-ALZ01 exhibited reduced expression of CD11b and GFAP in male and female SD rats compared to the LPS-only group. **(B-C)** Quantification of results demonstrated significant decrease in the expression CD11b and GFAP, compared to the LPS-only group, in the cortex of male (CD11b: ****p < 0.001; GFAP: **P < 0.01) and female (CD11b: ****p < 0.001; GFAP: ****p < 0.001) SD rats. **(D)** T-ALZ01 showed a decrease in the expression of CD11b and GFAP in the hippocampus of male and female SD rats compared to the LPS-only group. **(E-F)** Quantification of results showed significant reduction of the CD11b and GFAP in the hippocampus of male (CD11b: **p < 0.01; GFAP: ****p < 0.001) and female (CD11b: **p < 0.01; GFAP: ***p < 0.005) SD rats. The merged panels **(A, D)** illustrate the complete overlap of CD11b labelling and the DAPI stain (n=3; scale =200 μm).

To establish that T-ALZ01 also decreases neurodegeneration and the activation of microglia and astrocytes *in vivo*, immunofluorescent labeling on mid-sagittal hemisections of brain tissue was performed. SD brain tissue were fixed, equilibrated, and then embedded for sectioning at 14 μm (12–15 slices per mid-sagittal cut). The tissue sections were labelled with an anionic dye (green; degenerating neurons) or anti-CD11b (green; microglia) and anti-GFAP (red; astrocytes) antibodies as well as the nuclei stain, DAPI (blue). In control, untreated male SD rats (n = 4), low expression levels of CD11b, GFAP and the anionic dye were observed, compared to the LPS-only group (****p < 0.001). T-ALZ01 demonstrated significant decrease of the expression of the anionic dye, compared to the LPS-only group, in the cortex (****p < 0.001) and hippocampus (*p < 0.05) of male SD rats (Fig. 5A–D, respectively). Similarly, female SD rats showed significant decline in the expression of the anionic dye in the cortex and hippocampus (****p < 0.001) (Fig. 5A–D, respectively). T-ALZ01 also demonstrated lower expression levels of CD11b compared to the LPS-only group in the cortex (****p < 0.001) and hippocampus (****p < 0.001) of male rats (Fig. 6E–F). Female SD rats also exhibited less expression of CD11b after T-ALZ01 treatment in the cortex (****p < 0.001) and hippocampus (**p < 0.01) (Fig. 6A–F). GFAP expression levels were similarly reduced significantly in the cortex (**p < 0.01) and hippocampus (****p < 0.001) of male SD rats (Fig. 6A–F). Likewise, female SD rats showed lowered GFAP expression following T-ALZ01 treatment in the cortex (****p < 0.001) and hippocampus (***p < 0.005) (Fig. 6A–F). Thus, T-ALZ01 strongly reduces inflammation *in vivo* and exhibits efficacy in male and female SD rats.

## Discussion

The aim of this research was to highlight the therapeutic ability of T-ALZ01, delivered by microglia-derived extracellular vesicles called exosomes, in the alleviation of neuroinflammation in Alzheimer’s disease (AD). It was hypothesized that T-ALZ01, based on its mechanism of action, would inhibit the inflammatory processes that lead to neurodegeneration. First, the therapeutic effect of T-ALZ01 was examined *in vitro* through the analysis of inflammatory cytokines and complement pathway activity. This was followed by the analysis of T-ALZ01 loaded exosomes *in vivo* in LPS-induced neuroinflammation rat model to determine its effectiveness in the brain microenvironment. The complement system pathway was considered an important target as it is a major contributor to the inflammatory processes that cause neurodegeneration [19], [20], [23]. The complement protein inhibitors that have been tested in inflammatory diseases such as FUT-175, C3b inhibitor, C5a inhibitor and ANX-M1 (C1q inhibitor) have shown efficaciousness in the treatment of such diseases but also show detrimental side effects [18], [25].

The results of this study showed that the administration of T-ALZ01 in SD microglia cells demonstrated effective inhibition of C1r in a dose-dependent manner, as similarly demonstrated by Rushing et al. [26]. However, the concentrations of T-ALZ01 that did not completely inhibit C1r showed reduced neuroinflammation which is corroborated by Hong et al. whereby low doses of a C1q inhibitor were utilized to reduce neuroinflammation [25]. The analysis of the inflammatory cytokine levels *in vitro* and *in vivo* showed that T-ALZ01, compared to donepezil (DPZ), resulted in a significant reduction of pro-inflammatory cytokines (TNFα, IL-6). These cytokines are common markers of neuroinflammation as their mechanism of action exacerbates the macrophagic activity of the microglia further leading to astrogliosis and neuronal damage. In addition, the anti-inflammatory cytokine (IL-10) demonstrated increased protein levels in treated groups in the brain tissue, which symbolizes a more homeostatic brain environment [30], [31]. In contrast to SD microglial cells, cytokine analysis of an immortalized mouse microglial cell line (SIM-A9) showed increased sensitivity to T-ALZ01 and thus, did not demonstrate effective reduction of cytokine levels. These results are possibly due to the structural differences in the C1r protein and the low intrinsic activity of the complement pathway in mouse microglia cells compared to rat microglia cells [32]–[34]. Unlike rat, and human, the C1r protein in mice are composed of two isoforms (C1r-a and C1r-b) and thus, the use of T-ALZ01 could potentially affect the complement pathway in mice greater than rat, and human.

It has been reported that females are more prone to the development of AD compared to males [1], [2]. Females are exposed to more AD-associated risk factors such as stress, menopause-related hormone deprivation and diabetes, and thus have a more active inflammatory complement system compared to males [35]–[37]. As demonstrated by the results in the study, inflammatory dysregulation was stronger in females than males. Few animal studies have utilized females in the analysis of the efficacy of possible AD therapeutics. The ability of T-ALZ01 to alleviate neuroinflammation in the female groups would prove beneficial in the treatment of AD in a susceptible population. The efficacy of T-ALZ01 at a wider dosing range and different dosing regimen was not evaluated in this study, hence future research could analyze these conditions in male and female groups. Analysis of the long-term effect of T-ALZ01 would also prove beneficial in understanding its effect in both genders. Females are more sensitive to T-ALZ01 and thus, long-term inhibition of the C1r protein may pose substantial effects.

Though many complement protein inhibitors have been developed in the treatment of various neuroinflammatory diseases, the inability of these compounds to cross the blood-brain barrier (BBB) has limited usage. Unlike research conducted by Hong et al. where intravenous administration of a C1q inhibitor was conducted, this study utilized nanosized extracellular vesicles called exosomes for intranasal drug administration [25]. Similar to a study conducted by Haney et al., the biocompatible, pharmacokinetic, and targeted ability of exosomes proved beneficial in ensuring successful delivery of the candidate drug to the brain [38]. Exosomes, derived from healthy SD microglia, can travel to the microglia in the brain without being rejected by the immune system. However, off-targets to other resident brain cells are possible. The use of exosomes in the delivery of complement protein inhibitors would prove beneficial as a non-invasive method of treatment. The potential ease of administration would benefit the patient and doctor. Though the study utilized microglia-derived exosomes, it provides possible avenues that could be considered in drug delivery. Astrocyte-derived exosomes could be a potential candidate for the delivery of therapeutics to astrocytes, another contributor of neuroinflammation. Exosome-liposome hybrids would provide use of the benefits of both vesicles – improved targeted ability, biocompatibility, and drug loading [29], [39].

In summary, the study underscores the significant contribution of T-ALZ01 in the treatment of neuroinflammation in AD. Mechanistically, low concentrations of T-ALZ01 alleviate neuroinflammatory conditions without complete inhibition of the CCP2-SP domain of the C1r protein in male and female rats. Exosomes provide a non-invasive, biocompatible, and targeted alternative for the delivery of AD therapeutics to the brain. These findings provide insights into the potential therapeutic use of T-ALZ01 in mitigating the neuroinflammation associated with neurodegenerative diseases such as AD.

## Materials & Methods

### Materials

#### General reagents:

Dulbecco’s Modified Eagle’s Medium (DMEM) was purchased from Corning Life Sciences (Glendale, AZ, USA). Fetal Bovine Serum (FBS) was obtained from R&D Systems (Minneapolis, MN, USA). Goat Serum (GS) was obtained from Atlanta Biologicals (Flowery Branch, GA, USA). Phosphate Buffer Saline (PBS), Dimethyl Sulfoxide (DMSO), Sodium Chloride, Bovine Serum Albumin (BSA) and Penicillin/Streptomycin solution (PS) were purchased from VWR International (West Chester, PA, USA). The Acridine Orange/Propidium Iodide stain was purchased from Logos Biosystem (Annandale, VA, USA). VectaShield Mounting Medium with DAPI was purchased from Vector Laboratories, Inc. (Burlingame, CA, USA). Poly-D-lysine hydrobromide (PDL), Triton X-100 and Tween 20 were obtained from Sigma Aldrich (St. Louis, MO, USA). 4% Paraformaldehyde solution (PFA), Coumarin 6 and SuperSignal West Pico Chemiluminescent Substrate were procured from ThermoFisher Scientific (Waltham, MA, USA). MTT (3-(4,5-dimethylthiazol-2-yl)-2,5-diphenyltetrazolium bromide) was obtained from AmBeed (Arlington Heights, IL, USA). RIPA Lysis and Extraction Buffer was purchased from G Biosciences (St. Louis, MO, USA). PMSF (phenylmethansulfonyl fluoride) was obtained from Merck (Rahway, NJ, USA). Polyethylene glycol (PEG) (MW 6000) and LPS E. coli O55:B5 were acquired from Sigma Aldrich (St. Louis, MO, USA). LPS E. coli O8:K27 was purchased from Innaxon (Tewkesbury, UK). Tissue Plus OCT (optimal cutting temperature) compound, Tris-base, Sucrose, EZ-Run Pre-stained Rec Protein Ladder and Glycine were obtained from Fisher (Hampton, NH, USA).

### Antibodies

C1r mouse monoclonal antibody (mAb) (#sc-514105), goat anti-rabbit IgG-HRP (#sc-2004) and goat anti-mouse IgG-HRP (#sc-2005) were obtained from Santa Cruz Biotechnology (Dallas, TX, USA). CD11b/c mouse mAb (ab1211) was purchased from Abcam (Waltham, MA, USA). CD11b rabbit polyclonal antibody (pAb) (#NB110–89474) and GFAP rabbit pAb (#NB300–141) were procured from Novus Biologicals (Centennial, CO, USA). GAPDH rabbit mAb (#2118S) was purchased from Cell Signaling Technology (Danvers, MA, USA). PathoGreen Histofluorescent stain (#80027–5) was purchased from Biotium (Fremont, CA, USA). Cy3-goat anti-rabbit IgG (#111–165-144) and Cy2-goat anti-mouse IgG (#115–225-146) were procured from Jackson ImmunoResearch Laboratories (West Grove, PA, USA).

### Drugs

Donepezil (458050010) and TALZ-01 (S556–005) were obtained from ThermoFisher Scientific and ChemDiv respectively. For *in vitro* assays, both drugs were dissolved in double-distilled, sterile water. For *in vivo* assays, donepezil (DPZ) and TALZ-01 were dissolved in PBS.

### Animals

All animal experimental protocols and guidelines were approved by the St. John’s University Institute Animal Care and Use committee (IACUC Protocol #2035). Wild-type Sprague Dawley (SD) rats (9–10 weeks old) were treated in strict accordance with recommendations in the Guide for the Care and Use of Laboratory Animals of the National Institute of Health. The study was reported in accordance with ARRIVE guidelines. The animals were housed in a pathogen-free facility at 22 ± 2°C, 50 ± 5% humidity and 12-hour light/dark cycle with free access to chow diet and water. Males and females were housed separately.

### Cell viability analysis

The Sprague Dawley (SD) microglial cells were seeded in 96 well plates at 1 × 10^5^ cells/mL, with a final volume of 100 μL per well, and incubated at 37°C in 5% CO_2_ for 1 hour. The microglial cells were divided into two groups: (1) Control - cells were incubated with DPZ and T-ALZ01 (5–500 μM) and (2) Treated - cells were exposed to 100 ng/mL LPS for 30 minutes followed by the addition of DPZ and T-ALZ01 at a range of 10–100 μM. The cells were incubated at 37°C in 5% CO_2_ for 72 hours. After 72 hours, supernatant was discarded and 100 μL of 0.5% MTT solution was added to the 96-well plates and incubated for 2 hours. After dissolving the formazan product crystals in 100 μL of DMSO for 10 minutes at 37°C, the absorbance at 570 nm was measured using the Molecular Devices FilterMax F5 microplate reader. For Group 1, the viability of the untreated cells was set as 100%. For Group 2, the viability of the LPS treated cells was set as 100%. IC_50_ was calculated using the log inhibition curve generated through GraphPad Prism 9 software. The cell viability assay was performed in triplicate and each assay was run three times.

### Cytokine and complement activity analysis

The primary SD microglial cells were seeded at 1×10^5^ cells/mL in 24-well plates, with a final volume of 500 μL per well. Next, 100 ng/mL LPS was added, and the cells were further incubated for 30 minutes. The cells were then incubated with DPZ (50 μM) or TALZ-01 (10–50 μM) for 72 hours. The supernatant was collected and used in the following ELISA according to manufacturer instructions: (1) cytokine analysis - TNF-α, IL-6 and IL-10 from Boster Biological Technology (Pleasanton, CA, USA) and, (2) complement pathway activity – CH50 and C4 from AFG Scientific (Northbrook, IL, USA). Three independent experiments were conducted in duplicate. The generated standard curve was used to determine the concentration of each cytokine and complement protein with GraphPad Prism 9 software.

The detection and quantification of inflammatory cytokines and complement pathway activity in the hippocampal and cortical homogenates were conducted using ELISA according to manufacturer instructions from AFG Scientific (Northbrook, IL, USA). Three independent experiments were conducted in duplicate. The generated standard curve was used to determine the concentration of each cytokine and complement protein with GraphPad Prism 9 software.

### Preparation of cell lysates and tissue homogenates

The primary SD microglial cells were first seeded at 1×10^5^ cells/mL in a T25 flask. Next, 100 ng/mL LPS was added, and cells were incubated for 30 minutes. The cells were then exposed to 10, 30 and 50 μM of T-ALZ01 and incubated at 37°C in 5% CO_2_ for 24 hours. After 24 hours, the culture medium was aspirated and 3 mL of 1X PBS was added to the flasks. The cells were scraped, collected in a 15 mL centrifuge tube, and pelleted at 500×g for 10 minutes at 4°C. The supernatant was discarded and 50 μL 1X RIPA lysis buffer, supplemented with 1 mM PMSF, was added to the pellet. The resuspended cells were transferred to labelled microcentrifuge tubes and kept on ice for 30 minutes. The cells were then centrifuged at 12000 rpm for 20 minutes at 4°C. After 20 minutes, cell debris was removed, and the cell lysates were stored at −80°C for future western blot analysis. Protein concentrations were determined using bicinchoninic based (BCA) protein assay from ThermoFisher Scientific (Waltham, MA, USA).

The isolated cortex and hippocampus from one of the mid-sagittal hemisections (stored at −80°C) were thawed and weighed prior to the homogenization process. For every 0.5 g of tissue, 4 mL of the Homogenization Buffer (20 mM HEPES/0.32 M sucrose) in double-distilled water was added. At the time of homogenization, 1 mM PMSF was added. The homogenate was transferred to 15 mL tubes and spun for 5 minutes at 1000×g. The supernatant was transferred to a new 15 mL tube and spun for 20 minutes at 13300×g. The supernatant (S2 fraction) was transferred to a new 15 mL tube. The pellet (P2 fraction) was resuspended in P2 buffer at a volume equal to the initial weight of the tissue. Protein assays were carried out on each fraction. Each fraction was stored at −80°C.

### Western blot analysis

Whole cell lysates or tissue homogenates (50 μg) were diluted with 4X Laemmli sample buffer to final 1X dilution and western blot procedure was conducted according to Najafi et al. [40]. The C1r primary antibody (1:1000) and the HRP-conjugated secondary antibody (1:1000) were used. The HRP signals on the blots were developed using the SuperSignal West Pico Chemiluminescent Substrate and analyzed by capturing the chemiluminescence signal using the Omega Lum^™^ G Imaging System (Rockford, IL, USA). Quantification of the western blots were carried out using ImageJ (Image Processing and Analysis in Java 1.8.0_112) developed at NIH.

### Immunolabelling

In a 24-well cell culture plate, glass coverslips (12mm/Fisher Scientific, Pittsburgh, PA) were coated for 1 hour at room temperature with 500 μL poly-D-lysine (PDL at 0.1 mg/mL/0.1M boric acid). Next, the coverslips were washed 4 times with cell culture-grade distilled water (Millipore, MA, USA) and sterilized under UV light for 30 minutes. Primary SD microglia cells were seeded onto the PDL-coated coverslip at a density of 1×10^5^ cells/mL. The cells were then exposed to 100 ng/mL LPS. The cells were then treated with DPZ (50 μM) or T-ALZ01 (10, 30 and 50 μM) for 72 hours. The primary cells were then fixed and prepared according to Habeeb et al. [41]. The coverslips were incubated in the primary antibody (1:200) followed by the Cy3-conjugated secondary antibody (1:500) and then mounted on Fisherbrand glass microscope slides with a drop of VectaShield Mounting Medium with DAPI. Slides were stored at 4°C and sealed with nail polish the next day to prevent drying of the samples.

One of the mid-sagittal hemisections of the brains isolated from SD rats were fixed in ice cold 4% PFA/PBS in a 50 mL tube, with gentle agitation, for 2 hours at 4°C. Next, the brain tissue was transferred to 30% sucrose in PBS and incubated overnight at 4°C, with occasional gentle inversion of the tube. Once tissue was fully equilibrated, the brain tissue was embedded in OCT and then dredged in a 1:1 mixture of OCT and 30% sucrose until it was fully covered. The mold was then placed in a flat bed of pulverized dry ice and once frozen, the tissue block was wrapped in parafilm and stored at −80°C. One hour before sectioning, the tissue blocks were allowed to equilibrate in the Microm HM 525 cryostat microtome (Waltham, MA, USA). The fixed brain tissues were sliced at 14 μm (12–15 slices per mid-sagittal cut). The sections were then dipped into PBS to hydrate the slides and excess was dabbed off. The sections were blocked with 10% heat inactivated goat serum (HIGS) in PBT for 1 hour at room temperature then dipped once in PBT and excess was dabbed off. Following the blocking, the sections were stained with the primary antibodies (GFAP 1:1000, CD11b 1:500) in antibody dilution (100 μL HIGS/PBT) at 4°C overnight. After washing 3x with PBT for 5 minutes each, the tissues were incubated with fluorochrome-conjugated secondary antibodies (Cy2 anti-mouse 1:200, Cy3 anti-rabbit 1:500) in antibody dilution for 1 hour at room temperature. The immunostained tissue was washed 3x with PBT for 5 minutes then the VectaShield mounting solution with DAPI (Burlingame, CA, USA) was added and coverslip was placed. The slides were stored at 4°C until imaging.

### Exosome isolation and quantitation

FBS was spun at 100,000×g for 2 hours to remove exosomes before being added to the DMEM. SD microglial cells were then plated (1:25) in T225 flasks in media containing regular FBS (Control) or media containing exosome-depleted FBS (EXO-Dp) for 5–7 days. The vesicle-containing medium from cell culture was collected in 50 mL centrifuge tubes and exosomes were isolated according to Rider et al. [42]. Samples were centrifuged in a Beckman Coulter Allegra X-30R centrifuge (Jersey City, NJ, USA) at 4255×g for 1 hour at 4°C. The resulting pellet was suspended in 500 μL of PBS. Protein concentrations were determined using bicinchoninic based (BCA) protein assay from ThermoFisher Scientific (Waltham, MA, USA).

The control and EXO-Dp exosome samples (stored at −80°C) were thawed and gently vortexed before use in quantitation experiments analyzing fluorescence intensity and acetylcholinesterase activity. Coumarin 6 was used to analyze fluorescence intensity in exosome samples. Coumarin 6 powder was added to 200 μL of sample in a light-sensitive microcentrifuge tube and rocked overnight on a shaker at 37°C. The samples were then centrifuged at 6000 rpm for 2 minutes. Next, 50 μL of the supernatant was added to 450 μL of acetonitrile and mixed. Then, 50–100 μL was added to each well of a light-sensitive 96-well plate and read at 450 nm using the Molecular Devices FilterMax F5 microplate reader. Acetylcholinesterase activity was analyzed using the EXOCET Exosome Quantitation Kit from System Biosciences (Palo Alto, CA, USA). Instructions were followed according to the manufacturer. Experiments were carried out in duplicate from three independent experiments.

### Exosome characterization

The control and EXO-Dp exosome samples (stored at −80°C) were thawed and gently vortexed before quantification using the Exo-Check Antibody Array from System Biosciences (Palo Alto, CA, USA). Each array is comprised of 12 pre-printed spots and features 8 antibodies for known exosome markers (CD63, CD81, ALIX, FLOT1, ICAM1, EpCAM, ANXA5 and TSG101), a GM130 cis-Golgi marker for cellular contamination, two positive controls and a blank control. Instructions were followed according to the manufacturer. The arrays were developed using the SuperSignal West Pico Chemiluminescent Substrate and analyzed using the Omega LumTM G Imaging System (Rockford, IL, USA). Particle size analysis was conducted through dynamic light scattering (DLS) using the Zetasizer instrument (Malvern Panalytical). Samples were diluted in sterile, particle-free PBS at ratios of between 1:250 and 1:1,000 for optimum analysis. PBS was tracked before each experiment to ensure that it was particle-free. Cryo-TEM electron microscopy was conducted on exosomes (CUNY Advanced Research Center, NY, USA). Samples were first cryo-fixed (plunge-freeze) to eliminate water crystals that can affect structure, thus creating vitreous ice. Next, samples were transferred to the TEM for imaging. Approximately 10 images were taken of each sample.

### Exosome drug loading

T-ALZ01 was loaded into exosomes through sonication. T-ALZ01 (0.2 mg) was added to 200 μL of exosomes (~ 1×10^8^ exosomes) and the mixture was sonicated using a Cole-Parmer Ultrasonic Processor (Vernon Hills, IL, USA). The sonication settings used were 500 v, 2 kHz, 20% power, 6 cycles by 4 seconds pulse and 2 seconds pause according to Haney et al. [38]. The mixture was allowed to cool on ice for 2 minutes, and then the sonication cycle was repeated. The success of the drug loading was analyzed using high performance liquid chromatography (HPLC).

### Lipopolysaccharide (LPS)-induced neuroinflammation rat model

Eighteen male and eighteen female (9–10 weeks old) wild-type SD rats were purchased from Taconic Biosciences (Germantown, NY, USA). They were randomly and equally allocated to three treatment groups (n = 12/group) – PBS, DPZ and T-ALZ01. All animals received intraperitoneal injection of 0.5 mg/kg LPS daily. Four hours after each daily LPS injection, animals received treatment based on their allocated group. DPZ was administered orally at 1 mg/kg daily. T-ALZ01 was administered intranasally at 0.2 mg/kg. The animal experiment was carried out for 7 days. The rats were weighed daily and checked for any signs of toxicity or distress such as lethargy, loss of appetite and motor/behavioral changes (ataxia or lack of self-grooming). Observations were also made on the number of fecal pellets produced and reported. Once the experiment was completed, animals were sacrificed in 24–48 hours. Animals were exposed to 2% isoflurane for 2 minutes followed by sacrificial decapitation. The brains were isolated, and the brains were hemisected down the mid-sagittal plane. One hemisection was subjected to cryosectioning for use in immunocytochemistry and the second hemisection was used for biochemical and protein analysis. The brains of four untreated male SD rats (4 months old) were isolated and used as background control.

### Imaging

Fluorescent images were obtained using a Zeiss Axioplan 200M upright fluorescent microscope, AxioCam digital camera and AxioVision 4.8.2.0 software. Phase contrast images were collected using a Zeiss Axiovert 25CFL inverted microscope equipped with a Luminera Infinity 3 – 1 CCD camera and Infinity capture software (version 6.5.4).

### Statistical analysis

Significance is defined as p ≤ 0.05. Levels of significance are identified as follows: * p ≤ 0.05, ** p ≤ 0.01, *** p ≤ 0.001 and **** p ≤ 0.0005. All statistical analyses were conducted using GraphPad Prism 9 (San Diego, CA, USA). Unpaired two-tailed t-tests were used to compare two groups; to compare more than two groups, one-way analysis of variance (ANOVA) Tukey test was performed.

## Data Availability

All data generated or analyzed during this study are included in this article and its supplementary information files.
